# Implantable device with magnetically rotating disk for needle‐free administrations of emergency drug

**DOI:** 10.1002/btm2.10479

**Published:** 2023-02-01

**Authors:** Cho Rim Kim, Jae Hoon Han, Min Ji Kim, Myoung Ju Kim, Se‐Na Kim, Yong Chan Cho, Han Bi Ji, Chang Hee Min, Cheol Lee, Young Bin Choy

**Affiliations:** ^1^ Interdisciplinary Program in Bioengineering College of Engineering, Seoul National University Seoul Republic of Korea; ^2^ Institute of Medical and Biological Engineering, Medical Research Center, Seoul National University Seoul Republic of Korea; ^3^ Department of Pathology Seoul National University College of Medicine Seoul Republic of Korea; ^4^ Department of Biomedical Engineering Seoul National University College of Medicine Seoul Republic of Korea

**Keywords:** emergency drug, implantable device, magnetic actuation, needle‐free drug administration, on‐demand drug administration

## Abstract

Prompt administration of first‐aid drugs can save lives during medical emergencies such as anaphylaxis and hypoglycemia. However, this is often performed by needle self‐injection, which is not easy for patients under emergency conditions. Therefore, we propose an implantable device capable of on‐demand administration of first‐aid drugs (i.e., the implantable device with a magnetically rotating disk [iMRD]), such as epinephrine and glucagon, via a noninvasive simple application of the magnet from the outside skin (i.e., the external magnet). The iMRD contained a disk embedded with a magnet, as well as multiple drug reservoirs that were sealed with a membrane, which was designed to rotate at a precise angle only when the external magnet was applied. During this rotation, the membrane on a designated single‐drug reservoir was aligned and torn to expose the drug to the outside. When implanted in living animals, the iMRD, actuated by an external magnet, delivers epinephrine and glucagon, similar to conventional subcutaneous needle injections.

## 1 INTRODUCTION

Emergencies caused by life‐threatening medical events, such as anaphylaxis or hypoglycemia, often occur in community settings where healthcare professionals are not present.^1^, ^2^, ^3^, ^4^ Anaphylaxis, a potentially fatal allergic reaction, has been reported to affect up to 5% of the US population, with a 1% mortality risk.^5^, ^6^ Hypoglycemia is defined as an abnormally low plasma glucose level,^7^, ^8^ which is more relevant in patients with type 1 diabetes,^9^ accounting for a relevant death rate of more than 2%.^10^, ^11^, ^12^

In such emergency situations, there is a golden time for prompt drug administration to prevent a fatal event of death and increase the survival rate.^13^ Therefore, for the treatment of anaphylaxis and hypoglycemia, immediate administration of first‐aid drugs, such as epinephrine^14^, ^15^, ^16^ and glucagon,^10^, ^17^ is recommended when the patient first perceives the symptoms. For rapid systemic exposure, these drugs are prescribed to be administered by needle self‐injections,^13^, ^17^ where the patients need to be equipped with a prefilled syringe or kit to be filled with drugs,^18^, ^19^ and properly trained for injections at all times.^20^ However, the symptoms of anaphylaxis and hypoglycemia often include blocked breathing, dizziness, or trembling,^21^, ^22^ which makes self‐injections difficult.^19^ When involved with needle phobia, which is reported to interfere with 7%–22% of patients prescribed needle self‐injections,^15^, ^23^, ^24^ the chances of proper drug injections could be lower under such emergency conditions.

As a result, an implantable device with on‐demand drug delivery may be advantageous as a life‐saving strategy in emergencies. The device is prefilled with drugs sufficient for multiple doses to be used with patients at all times after implantation. Moreover, when the symptoms are perceived, the patient can immediately respond to administering a first‐aid drug through a modality without needles.^25^, ^26^ However, in many previous devices, such on‐demand drug delivery was enabled with electronic actuators, circuits, and batteries embedded in the device,^27^, ^28^, ^29^ making it bulky and inconvenient for implantation. Considering fatal but infrequent emergency occurrences, the limited lifetime of the battery due to the discharge through the implemented circuit can be a disadvantage, which may require replacement surgery even before complete drug consumption.^27^, ^28^, ^29^, ^30^, ^31^

Therefore, we propose a battery‐less implantable device actuated solely by magnetic force for the on‐demand delivery of emergency drugs. Inside the device, there is a disk containing multiple drug reservoirs arranged at a precise angular spacing and hermetically sealed with a membrane. The disk contains a magnet that allows for the actuation of up and down movements by a magnetic force applied externally from the device. At each actuation, the disk can rotate at a precise angle along a guiding path, where the membrane of a specifically designated single reservoir can be aligned and torn by a pole formed in the device, thereby exposing and releasing the drug. This actuation is based on mechanical means enabled only when a magnet is applied from the outside skin, thereby on‐demand drug delivery without electronic power.

In this study, an implantable device with a magnetically rotating disk (iMRD) was assessed with epinephrine and glucagon, representative first‐aid drugs for anaphylaxis and hypoglycemia, respectively. The drug‐loaded iMRD was implanted subcutaneously in rats, and a magnet was applied from the outside skin at predetermined times to assess the capacity of on‐demand drug delivery. We examined the pharmacokinetic (PK) profile and compared it to that of conventional subcutaneous needle injections. For glucagon, the pharmacodynamic (PD) profile (i.e., change in plasma blood glucose level) was also monitored in hypoglycemia‐induced rats.

## 2 RESULTS

### 2.1 iMRD design and working principle

**FIGURE 1:**
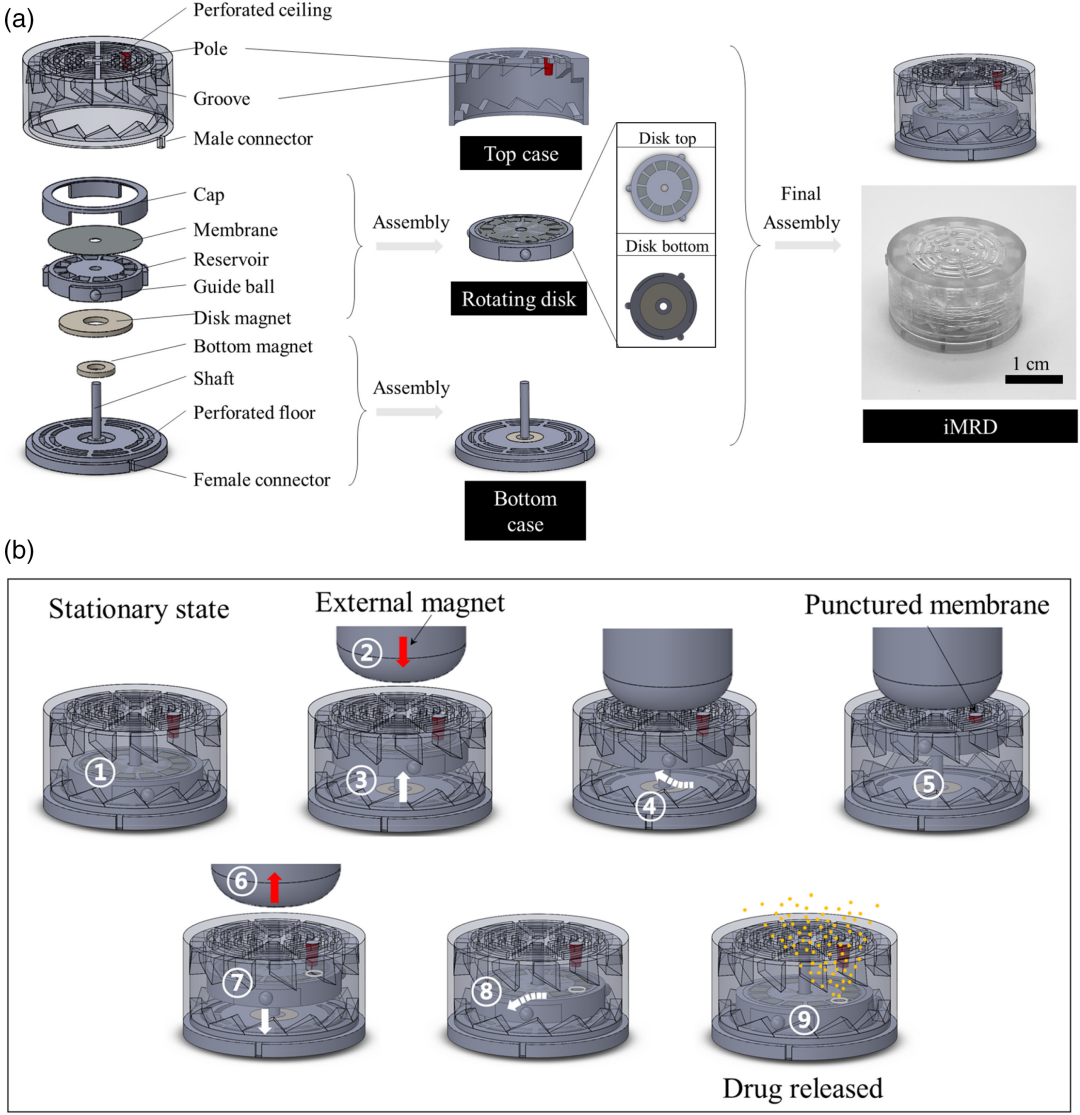
Schematic description of the implantable device with a magnetically rotating disk (iMRD). (a) Assembly procedure and (b) working principle.

The iMRD was prepared by assembling and bonding the constituent units, each of which was prepared by 3D printing, as shown in Figure 1a. In the rotating disk, 12 distinct drug reservoirs, each with a volume of approximately 13 μl, were equally spaced at an angle of 30°. Each reservoir contained an accurate amount of drug in dry form, which was measured as 980.13 ± 10.45 μg epinephrine or 19.20 ± 0.85 μg glucagon, respectively. After full assembly, the dimensions of the iMRD were 27 mm in diameter and 15 mm in height, giving a total volume of approximately 8.6 ml. Figure S1 shows optical images of the actual constituent units and assembly procedure.

Figure 1b depicts the detailed working principle of the iMRD (see Movie S1). The rotating disk is inserted into the shaft of the bottom case without bonding; thus, it is basically mobile. However, the disk magnet (0.16 T) and bottom magnet (0.14 T) face each other with opposite polarities, and because of their magnetic attraction, the disk is firmly attached to the bottom floor without movement in the stationary state (①). When a strong magnet (0.3 T) with an opposite polarity to the disk magnet approaches the iMRD (②), the attractive force between the disk and bottom magnets is overcome to pull the disk upward (③). During this movement, the guide balls in the rotating disk tilt the perpendicular movement through the grooves to make the disk rotate clockwise to a precise angle of 25° in the horizontal plane (④). When the disk reaches the ceiling, the pole at the ceiling is aligned and the membrane is punctured on a designated reservoir (⑤). When the external magnet is removed (⑥), the disk moves downward due to the attraction between the disk and bottom magnets (⑦), where the guide balls and grooves again make the disk rotate clockwise to a precise angle of 5° (⑧). Through a punctured membrane, the drug in the reservoir is released and exposed to the outside through the perforated ceiling and floor of the iMRD (⑨) while the disk is back to the stationary state. This process is repeated at every actuation to rotate the disk and puncture the membrane of the next designated reservoir.

### 2.2 In vitro performance tests

To examine the performance of on‐demand drug delivery, the iMRD, each loaded with epinephrine or glucagon, was fully submerged in PBS (pH 7.4) and actuated six times consecutively using an external magnet applied on the top of the iMRD at a 1 mm spacing to simulate the skin over the implanted device.^32^, ^33^ For both epinephrine and glucagon, almost all drug in a reservoir was dissolved and exposed to the outside of the iMRD quite rapidly, within minutes. As shown in Figure 1a,b, the amount of drug released per actuation was highly reproducible, which was measured as 980.13 ± 10.45 and 19.20 ± 0.85 μg per actuation for epinephrine and glucagon, respectively. After six actuations, the iMRD was disassembled to observe the rotating disk, where the six distinct holes made in the membrane over the designated reservoirs were clearly observed, while the rest remained intact (Figure S2 and Movie S2).

**FIGURE 2:**
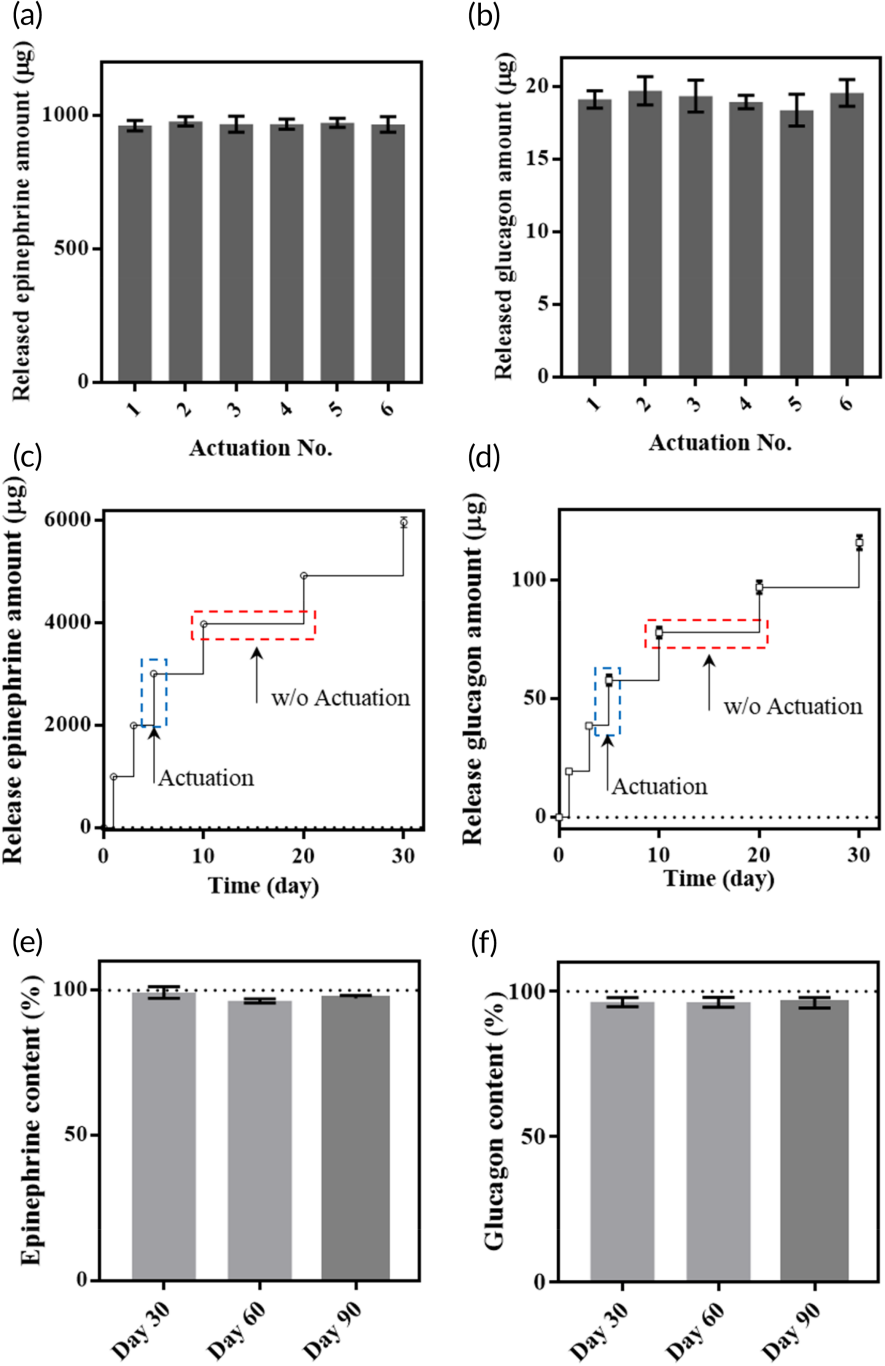
In vitro performance test of the device, which was performed while the implantable device with a magnetically rotating disk (iMRD) was fully immersed in pH 7.4 PBS at 37°C. The device was actuated six consecutive times, and the amount of newly released (a) epinephrine and (b) glucagon was measured after each actuation. Cumulative release amounts of (c) epinephrine and (d) glucagon were measured before and after the iMRD actuation at scheduled times of 1, 3, 5, 10, 20, and 30 days. For stability evaluation, (e) epinephrine and (f) glucagon were stored in the reservoir of the device at 37°C for 30, 60, and 90 days. After each incubation time, the content was measured using high‐performance liquid chromatography (HPLC). For each drug kind, and measurement and incubation time, the experiment was performed in triplicate. Data are presented in the form of mean ± standard deviation (SD).

When the iMRD was immersed in PBS for a long‐term period of 30 days, the drug was released only when the external magnet approached to actuate the iMRD, as shown in Figure 1c,d. No added drug was detected immediately before actuation, indicating that there was no leak or unexpected membrane breakage when the iMRD was in the stationary state. Considering the implantation, the stability of epinephrine and glucagon loaded in the iMRD was also tested under simulated biological conditions, that is, incubation at body temperature. As shown in Figure 2e,f, almost all the drugs retained their stability for up to 90 days. When glucagon after 90‐day incubation was injected into hypoglycemic animals, the increase in blood glucose level was not different from that of the animals injected with fresh glucagon; hence, there was retained biological activity (Figure S3).

### 2.3 In vivo performance tests

**FIGURE 3:**
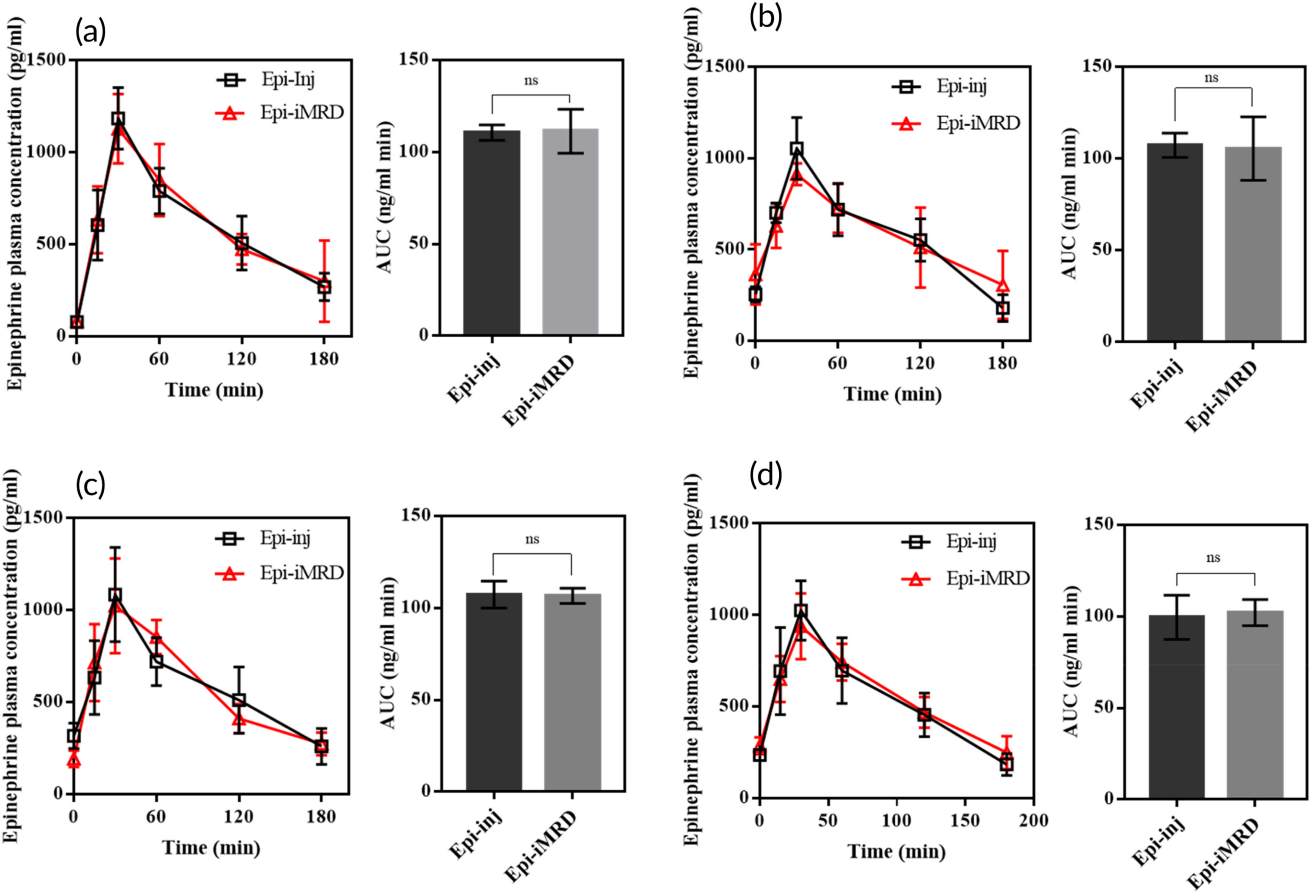
In vivo pharmacokinetic profiles of epinephrine obtained by subcutaneous injections (Epi‐inj; *n* = 4) and device actuations (Epi‐implantable device with a magnetically rotating disk (iMRD); *n* = 4) at (a) 15, (b) 30, (c) 45, and (d) 60 days after the onset day of experiments. The area under the plasma drug concentration–time curve (AUC) was calculated using the trapezoidal rule. Data are presented in the form of mean ± SD.

To assess the capacity of needle‐free delivery and prompt systemic exposure to drugs, an iMRD loaded with epinephrine, a representative emergency drug, was first tested under in vivo experimental conditions. For this, the iMRD loaded with epinephrine was subcutaneously implanted (i.e., Epi‐iMRD) for 45 days and actuated by a magnet at scheduled times, where the PK profile was compared with the animals subcutaneously injected with epinephrine (i.e., Epi‐inj). As shown in Figure 3 (Table S1), the PK profiles of both animal groups were similar on all scheduled days of drug administration (*p* > 0.05), showing a similar maximum plasma drug concentration (C_max_) and time to reach C_max_ (T_max_ = 30 min). Thus, the area under the plasma drug concentration–time curve (AUC) was also similar between the two groups (*p* > 0.05). In this study, the drug was administered at a relatively wide time interval of 15 or more days for a total of 60 days, and it should be noted that the PK profile was reproducible at all administration times with the iMRD.

**FIGURE 4:**
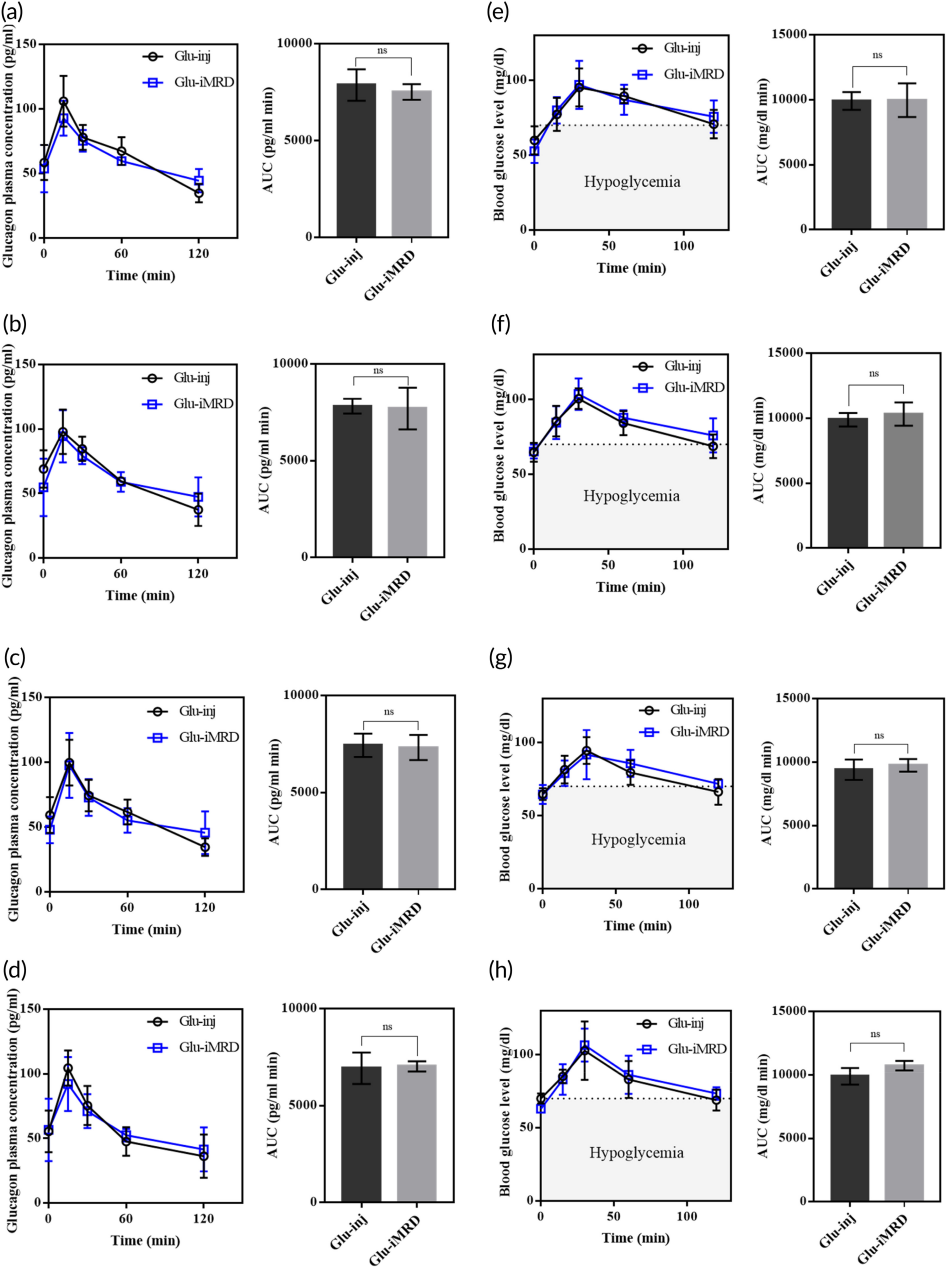
In vivo (a–d) pharmacokinetic and (e–h) pharmacodynamic profiles of glucagon obtained by subcutaneous injections (Glu‐inj; *n* = 4) and device actuations (Glu‐iMRD; *n* = 4) at (a, e) 15, (b, f) 30, (c, g) 45 and (d, h) 60 days after the onset day of experiments. The area under the plasma drug concentration–time curve (AUC) was calculated using the trapezoidal rule. Data are presented in the form of mean ± SD.

When loaded with glucagon, another emergency drug, iMRD delivered the drug in a manner similar to subcutaneous injections. As shown in Figure 4a–d (Table S2), the PK profiles of Glu‐iMRD and Glu‐inj were quite similar, showing the same *T*_max_ (15 min) and similar C_max_ and AUC (*p* > 0.05). This PK profile was reproducible at all administration times using iMRD. For glucagon, we also examined the PD profile, that is, the profile of blood glucose levels, in hypoglycemia‐induced animals. As shown in Figure 4e–h, after actuation of the iMRD, the blood glucose level increased, indicating viable bioactivity of glucagon. Notably, the PD profile of Glu‐iMRD was quite similar to that of Glu‐inj at all administration times for 60 days, suggesting an effective hypoglycemic treatment similar to conventional needle injections.

### 2.4 Histology

**FIGURE 5:**
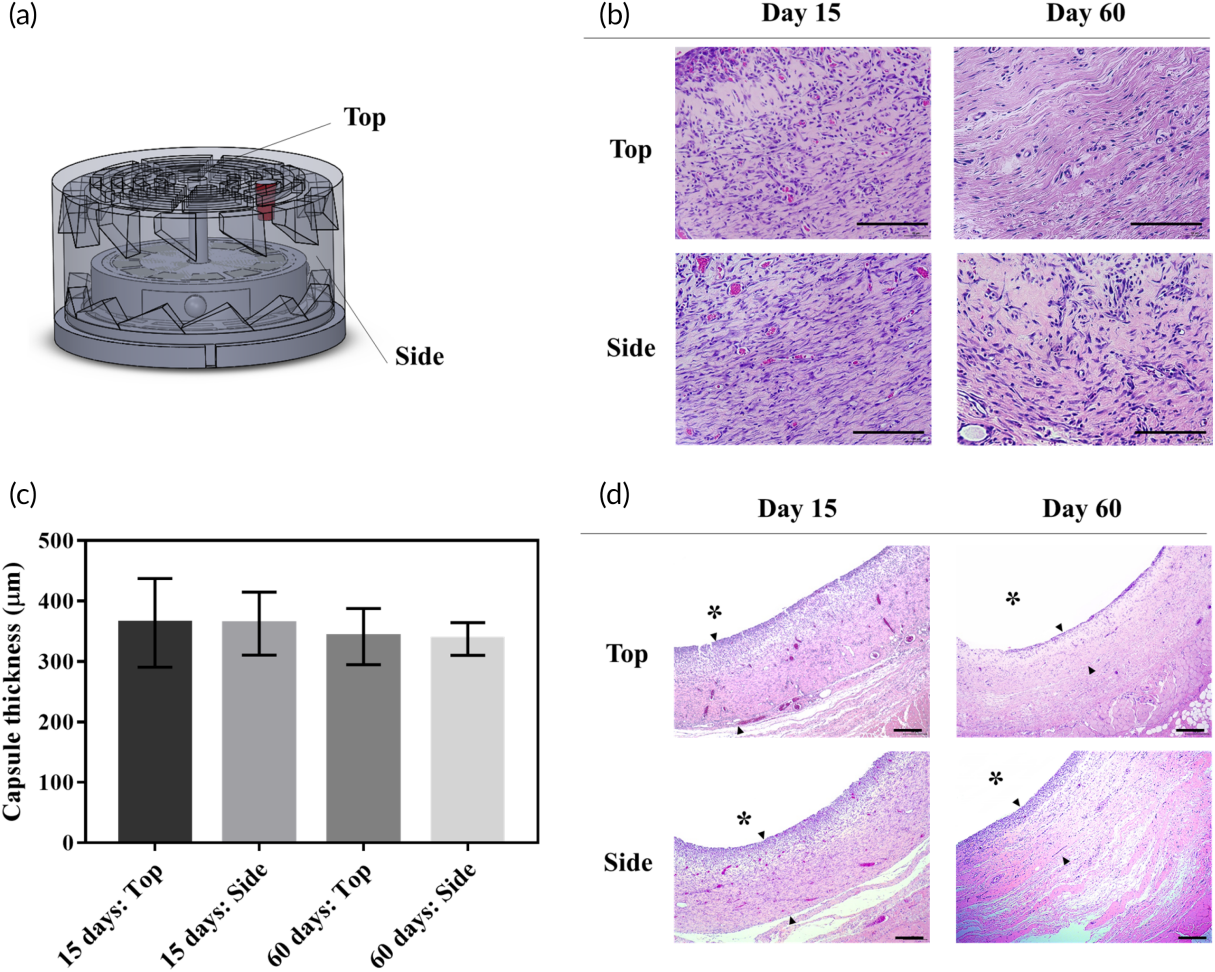
Histological analysis results. (a) Locations of the analyzed tissues surrounding the device. (b) Representative optical images of the hematoxylin and eosin (H&E)‐stained tissues biopsied at the ending point of experiments (60 days). The images were obtained at ×100 magnification. Scale bar, 100 μm. (c) Fibrotic capsule thicknesses formed around the device after 15‐ and 60‐day implantation (*n* = 4). (d) Representative optical images of the H&E‐stained tissues biopsied after 15‐ and 60‐day implantation. Asterisk indicates the location of the device. Arrows show the capsule thickness. The images were obtained at ×40 magnification. Scale bar, 100 μm.

To evaluate in vivo biocompatibility, the tissues surrounding the iMRD were biopsied at 15 and 60 days after implantation. In this study, we assessed H&E‐stained tissues near two different locations, that is, the top and side walls of the iMRD, which represent the tissues near and far from where an external magnet was applied, respectively (Figure 5a). Overall, the results of the histological analysis were not different between Epi‐iMRD and Glu‐iMRD. As shown in Figure 5c, a mild inflammatory response was observed in the periphery, which was not further upregulated as time elapsed after implantation. Figure 5d shows fibrotic capsules formed at both tissue locations, as reported for many other nondegradable implants.^28^, ^34^, ^35^ However, the thickness was not significantly different between the two locations (*p* > 0.05), suggesting no significant mechanical stress due to magnetic actuation. From 15 days after implantation, the capsule thickness stabilized without an increase, as observed in our previous studies.^36^, ^37^

## 3 DISCUSSION

In life‐threatening medical emergencies in community settings, such as anaphylaxis and hypoglycemia, prompt drug administration can actually save lives.^3^, ^15^, ^17^ These events require rapid systemic drug exposure, which is often achieved using needle injections. For this purpose, the needle‐based auto‐injectors are already commercially available,^38^, ^39^ where the individual needs to be well established and experienced with such a first‐aid injection plan without the presence of medical professionals.^40^, ^41^, ^42^ However, medical emergencies are often accompanied by symptoms that interfere with delicate movements, and needle injections are difficult to perform.^43^, ^44^

Therefore, we proposed an implantable device, that is, the iMRD, that could deliver a first‐aid drug of interest easily and noninvasively by a simple magnet application from the outside skin. The structure of the iMRD was carefully designed to provide on‐demand drug delivery capacity (Figure 1), and the use of magnets in an implantable device has already been adapted clinically, such as in cochlear implants.^45^ Multiple drug reservoirs were hermetically sealed with a biocompatible titanium membrane^46^, ^47^; therefore, no drug was released in the absence of magnetic applications (Figure 2c,d). These reservoirs were located at an accurate angular spacing on the disk, and the movement path of the disk was precisely guided through the grooves in the iMRD. Thus, only at the time of magnet application, the disk could rotate to accurately align and tear the membrane of a single designated reservoir (Figure S2 and Movie S2).

We pursued to obtain a similar PK profile between the groups of iMRD and subcutaneous injections. For this, we intentionally prepared the ceiling and floor of the iMRD to be perforated so that a bodily fluid could be readily in contact with the membrane. The drug in each reservoir was formulated to be a highly porous tablet of pure drug itself by freeze‐drying. Thus, right after the breakage of the membrane, the drug could be rapidly dissolved and exposed to the outside of the iMRD (<10 min), which allowed for prompt systemic exposure of the drug, as observed with subcutaneous needle injections (Figures 3 and 4).

The actuation of the IMRD was based solely on magnetic force without any other power sources, thereby eliminating batteries or electronics. This would allow for smaller dimensions and long‐term operation of the device after implantation compared to other implantable devices for on‐demand drug delivery.^48^, ^49^ The device size can be further reduced when fabricated in a more sophisticated manufacturing facility.^50^ The attraction force between the disk and bottom magnets (c.a. 0.12 N) was strong enough to prevent an accidental movement of the disk (weighing c.a. 2 g). Upon this setting, the iMRD herein was designed to actuate with a relatively strong magnetic field (>3000 G) applied at a distance greater than the thickness of human skins (Table S3), and this would again avoid any unexpected actuations caused by the household electronics (~2 G).^51^

Considering translational applications, the actuation device housing may need to be made of harder materials, such as metals, to better protect the drug in the device after implantation.^48^, ^52^ In our prototype, 12 separate drug reservoirs were used. The average incidence of anaphylaxis and severe hypoglycemia is reported to be once a year or once every 2 years,^53^, ^54^ implying that the iMRD in its current form can be envisioned for more than a 10‐year use after a one‐time implantation. In this aspect, an iMRD with a smaller number of reservoirs would still be useful; hence, a smaller device dimension. Unlike implantable drug delivery devices based on liquid pumping,^37^, ^55^, ^56^, ^57^ the iMRD releases the drug by opening a single reservoir, each filled with an accurate dose of drugs in dry form, which is expected to generally improve the drug stability for prolonged periods compared with that in liquids, especially for epinephrine and glucagon.^58^, ^59^, ^60^

## 4 CONCLUSIONS

We propose an implantable device for the simple and prompt administration of first‐aid drugs without needles in medical emergencies. The device is embedded with a disk containing multiple drug reservoirs hermetically sealed with a membrane, each of which can be opened only at the time of noninvasive magnet application from the outside skin. This actuation can be achieved by the precision engineering of the device, where the disk can rotate at an accurate angle through the guides formed in the device during actuation mediated by a magnetic force. The iMRD, therefore, exhibited rapid exposure to first‐aid drugs, such as epinephrine and glucagon, within minutes only at the time of an external magnet application. After implantation, the iMRD showed a similar profile of drug exposure and efficacy to subcutaneous injections. We therefore conclude that iMRD could be a promising strategy for the needleless prompt administration of first‐aid drugs during medical emergency events.

## 5 MATERIALS AND METHODS

### 5.1 Materials

Veroclear and SUP706, used for 3D printing, were purchased from Stratasys (Rehovot, Israel). The medical epoxy (Epo‐Tek 301) was obtained from Epoxy Technology (MA, USA). The magnets were obtained from Daehan Magnet (Seoul, Korea). Titanium membranes with a thickness of 2 μm were purchased from Nilaco (Tokyo, Japan). Acetonitrile (ACN) was obtained from Daejung (Siheung, Korea). Phosphate buffered saline (PBS) and formalin were purchased from Thermo Fisher Scientific (MA, USA). Epinephrine, trifluoroacetic acid (TFA), and acetaminophen were purchased from Sigma‐Aldrich (MO, USA). Glucagon was obtained from Novo Nordisk (Bagsværd, Denmark). The adrenaline/epinephrine enzyme‐linked immunoassay (ELISA) kit was purchased from MyBioSource (CA, USA). The rat glucagon enzyme immunoassay (EIA) kit was obtained from RayBiotech (GA, USA). Gentamicin was purchased from Shinpoong (Seoul, South Korea).

### 5.2 iMRD Fabrication

The major constituent units of the iMRD were designed using 3D CAD SolidWorks (Dassault Systemes, Vélizy‐Villacoublay, France) and fabricated using a 3D printer (Objet 30 Pro, Stratasys, Rehovot, Israel). As shown in Figure 1a (see Figure S1), the iMRD was composed of three main parts: a top case, rotating disk, and bottom case. In the top case, a groove for a guiding path was formed in the inner wall to allow for the rotation of the disk at a precise angle during its upward and downward movement. A pole was placed on the ceiling to puncture the membrane on the drug reservoir when the disk moved upward. The ceiling was perforated so that the drug exposed after membrane breakage could be released outside the iMRD.

Three guide balls were made at the side wall of the rotating disk (Figure S1) with an angular spacing of 120°, allowing the disk to slide and rotate through the tilted grooves made in the top case during the upward and downward movements. In the body of the disk, 12 individual drug reservoirs were prepared at an angular spacing of 30°, each of which was filled with 1000 μg of epinephrine or 20 μg of glucagon. To load the drug, 10 μl of an aqueous solution of epinephrine (100 mg/ml) or glucagon (2 mg/ml) prepared in sterile water was added to each reservoir, and the whole disk was exposed to liquid nitrogen and lyophilized for more than 6 h (FreeZone 6 dryer system; Labconco, USA). Immediately thereafter, the disk was seamlessly sealed with a titanium membrane using medical epoxy, which was then covered with a cap. A donut‐shaped magnet (0.16 T) was bonded to the bottom of the disk (i.e., the disk magnet). In the bottom case, a donut‐shaped magnet (0.14 T) was inserted into the shaft and bonded to the floor using medical epoxy (i.e., the bottom magnet). The floor was also prepared to be perforated to allow drug release to the outside. For the final assembly, the rotating disk was inserted into the shaft of the bottom case. Then, the top and bottom cases were aligned using male and female connectors and bonded using medical epoxy.

### 5.3 iMRD characterizations

To measure the actual drug‐loading amount, a drug‐loaded rotating disk without a membrane was fully immersed in 10 ml of phosphate‐buffered saline (PBS) at pH 7.4, which was sonicated for 10 min to completely dissolve the drug. The medium was then analyzed using high‐performance liquid chromatography (HPLC Agilent 1260 series; Agilent Technologies, CA, USA) equipped with a Diamonsil C18 column (150 × 4.6 mm, 5 μm; Dikma Technologies, USA). For epinephrine measurement, the mobile phase was prepared using a mixture of aqueous solutions of 0.1% TFA and ACN (v/v = 50:50). The absorbance wavelength and flow rate were set to 243 nm and 0.75 ml/min, respectively. For glucagon measurements, the mobile phase was a mixture of 0.05% TFA in DI water (TFA/DI) and 0.02% ACN in TFA (ACN/TFA). The mobile phase was pumped at a flow rate of 0.75 ml/min in the gradient mode, in which the volume ratio of TFA/DI:ACN/TFA was changed from 80:20 to 0:100 for 25 min. Glucagon concentration was detected at 214 nm.

To evaluate the in vitro performance of on‐demand drug delivery, the iMRD was fully immersed in 10 ml of PBS (pH 7.4) at 37°C, and actuated with a magnet (0.3 T) externally approaching the ceiling of the iMRD at a gap of 1 mm to mimic the presence of the skin layer.^32^, ^33^ Actuation was performed at scheduled times of 1, 3, 5, 10, 20, and 30 days. Immediately before and after each actuation, 5 ml of medium was extracted and replaced with fresh PBS. The collected medium was analyzed by HPLC as described above to measure the concentrations of epinephrine and glucagon.

### 5.4 Drug stability evaluation

Considering the drug stability after implantation, a reservoir was prepared as in the iMRD, which was filled with epinephrine or glucagon and sealed with the membrane. The drug‐containing reservoir was stored in an incubator at 37°C for 30, 60, and 90 days. After each incubation, the membrane was torn to expose the drug in the reservoir, which was fully immersed in 1 ml of PBS (pH 7.4) to dissolve the drug. The medium was collected and assessed by HPLC, as described above, and compared with that of a fresh drug without incubation. Experiments were performed in triplicate for each incubation time for epinephrine and glucagon.

### 5.5 Animal study

All in vivo experimental procedures were performed using Wistar rats (Charles River, Massachusetts, USA), weighing 180 ± 30 g, following institutional guidelines approved by the Institutional Animal Care and Use Committee at Seoul National University Hospital Biomedical Research Institute (approval no. 21‐0234‐S1A0). The animals were housed in a pathogen‐free facility with controlled temperature, humidity, and a 12:12 h light/dark cycle. Standard chow and water were provided ad libitum. Prior to implantation, all iMRDs were sterilized with ethylene oxide gas.^61^ To implant the iMRD, the animal was anesthetized by isoflurane inhalation, and the flank area was shaved and sterilized with betadine. A 33‐mm incision was made and the iMRD was implanted into the subcutaneous pocket. The incision was closed with surgical sutures and disinfected with betadine. Gentamicin (10 mg/kg) and acetaminophen (50 mg/kg) were then injected subcutaneously and intraperitoneally, respectively.

### 5.6 PK evaluation of epinephrine administration

For the PK test of epinephrine, the animals were divided into two groups: (1) Epi‐inj (*n* = 4): animals treated with subcutaneous injections of 500 μg epinephrine and (2) Epi‐iMRD (*n* = 4): animals implanted with the epinephrine‐loaded iMRD containing 1000 μg epinephrine in each reservoir. On the onset day of the experiment, the iMRD was implanted for the Epi‐iMRD, while the animals in the Epi‐inj group were left untreated. After 15, 30, 45, and 60 days, the drug was administered by subcutaneous needle injections and magnetic applications (0.3 T) from outside the skin above the implanted iMRD for Epi‐inj and Epi‐iMRD, respectively. At 0, 15, 30, 60, 120, and 180 min after each drug administration, 250 μl of blood was extracted from the tail vein and centrifuged at 3000 *g* for 10 min to collect 100 μl of plasma. The drug concentration in blood plasma was analyzed using an epinephrine/adrenaline ELISA kit.

### 5.7 PK and PD evaluation of glucagon

For the PK test of glucagon, the animals were divided into two groups: (1) Glu‐inj (*n* = 4): animals treated with subcutaneous injections of 10 μg glucagon; and (2) Glu‐iMRD (*n* = 4): animals implanted with glucagon‐loaded iMRD containing 20 μg glucagon in each reservoir. Drug administration and blood extraction were performed as previously described. A rat glucagon EIA kit was used to measure glucagon concentration in the blood plasma. For the PD test of glucagon, we measured the blood glucose level using a glucometer (Accu‐check Performa, Basel, Switzerland). Prior to each administration day, the animals were fasted overnight to induce hypoglycemia, which was confirmed by a blood glucose level of less than 70 mg/dl.^36^, ^62^ At 0, 15, 30, 60, 120, and 180 min after glucose administration, 1–2 μl of blood was collected from the tail vein and measured using a glucometer.

### 5.8 Histology

At 15 and 60 days post‐implantation, the animals in the Epi‐iMRD (*n* = 4) and Glu‐iMRD (*n* = 4) groups were sacrificed by carbon dioxide inhalation, and the tissue surrounding the iMRD was harvested from two distinct locations, that is, the top and side walls of the iMRD (Figure 5a). The tissue was then fixed in 4% paraformaldehyde and embedded in paraffin wax. The paraffin block was cut into slices of 4 μm thickness, among which three slides were randomly selected from each day and location in each animal to prepare tissue slides. The tissue was then stained with hematoxylin and eosin (H&E) to assess the degree of inflammation and fibrous capsule thickness around the iMRD, as described in our previous studies.^34^, ^63^ The analysis was performed by a professional pathologist (C.L.) in a blinded manner using an optical microscope at ×40 and ×100 magnification (Nikon, Eclipse Ci‐L, Tokyo, Japan).

### 5.9 Statistical analysis

Data are presented as mean ± standard deviation, and differences between groups were determined by two‐way analysis of variance (ANOVA) followed by post hoc Tukey's test for multiple comparisons. Student's t‐test was used to compare the means of the two groups. Statistical analysis was performed using GraphPad Prism 7 (GraphPad Software, CA, USA). Differences were considered statistically significant when *p* < 0.05.

## AUTHOR CONTRIBUTIONS

**Cho Rim Kim:** Conceptualization (equal); data curation (equal); formal analysis (equal); investigation (equal); methodology (equal); resources (equal); software (equal); validation (equal); visualization (equal); writing – original draft (equal); writing – review and editing (equal). **Jae Hoon Han:** Investigation (equal); methodology (equal). **Min Ji Kim:** Investigation (equal); methodology (equal). **Myeong Ju Kim:** Methodology (equal). **Se Na Kim:** Investigation (equal). **Yong Chan Cho:** Investigation (equal); methodology (equal). **Han Bi Ji:** Investigation (equal); methodology (equal). **Chang Hee Min:** Methodology (equal). **Cheol Lee:** Investigation (equal); validation (equal). **Young Bin Choy:** Funding acquisition (lead); investigation (equal); methodology (equal); project administration (lead); supervision (lead); writing – review and editing (lead).

## CONFLICT OF INTEREST

Cho Rim Kim and Young Bin Choy are listed as inventors on the granted patent (KR10‐2394358) filed by SNU R&DB for the implantable devices described in this article. The other authors declare no conflicts of interest.

## Supporting information


**Data S1:** Supporting Information

**Movie S1:** Working principle of the iMRD.

**Movie S2:** Membrane after iMRD actuations.

## Data Availability

Data are available from the authors upon reasonable request.

## References

[1] Simons FER , Organization, W. A . Epinephrine auto‐injectors: first‐aid treatment still out of reach for many at risk of anaphylaxis in the community. Ann Allergy Asthma Immunol. 2009;102(5):403‐409. 19492662 10.1016/S1081-1206(10)60512-1

[2] McLean‐Tooke AP , Bethune CA , Fay AC , Spickett GP . Adrenaline in the treatment of anaphylaxis: what is the evidence? Br Med J. 2003;327(7427):1332‐1335. 14656845 10.1136/bmj.327.7427.1332 PMC286326

[3] Lieberman P , Kemp SF , Oppenheimer J , et al. The diagnosis and management of anaphylaxis: an updated practice parameter. J Allergy Clin Immunol. 2005;115(3):S483‐S523. 15753926 10.1016/j.jaci.2005.01.010

[4] Elwen FR , Huskinson A , Clapham L , et al. An observational study of patient characteristics and mortality following hypoglycemia in the community. BMJ Open Diabetes Res Care. 2015;3(1):e000094. 10.1136/bmjdrc-2015-000094 PMC4486683 26157583

[5] Shaker M , Kanaoka T , Murray RG , Toy D , Shaker S , Drew A . A survey of caregiver perspectives on emergency epinephrine autoinjector sharing. J Allergy Clin Immunol Pract. 2018;6(5):1792‐1795. 29499333 10.1016/j.jaip.2018.02.017

[6] Turner PJ , Jerschow E , Umasunthar T , Lin R , Campbell DE , Boyle RJ . Fatal anaphylaxis: mortality rate and risk factors. J Allergy Clin Immunol Pract. 2017;5(5):1169‐1178. 28888247 10.1016/j.jaip.2017.06.031 PMC5589409

[7] Cryer PE , Fisher JN , Shamoon H . Hypoglycemia. Diabetes Care. 1994;17(7):734‐755. 7924788 10.2337/diacare.17.7.734

[8] Seyed Ahmadi S , Westman K , Pivodic A , et al. The association between HbA1c and time in hypoglycemia during CGM and self‐monitoring of blood glucose in people with type 1 diabetes and multiple daily insulin injections: a randomized clinical trial (GOLD‐4). Diabetes Care. 2020;43(9):2017‐2024. 32641374 10.2337/dc19-2606 PMC7440892

[9] Isaacs D , Clements J , Turco N , Hartman R . Glucagon: its evolving role in the management of hypoglycemia. Pharmacotherapy. 2021;41(7):623‐633. 33963599 10.1002/phar.2534

[10] Kedia N . Treatment of severe diabetic hypoglycemia with glucagon: an underutilized therapeutic approach. Diabetes Metab Syndr Obes. 2011;4:337. 21969805 10.2147/DMSO.S20633 PMC3180523

[11] Laing S , Swerdlow A , Slater S , et al. The British diabetic association cohort study, II: cause‐specific mortality in patients with insulin‐treated diabetes mellitus. Diabet Med. 1999;16(6):466‐471. 10391393 10.1046/j.1464-5491.1999.00076.x

[12] Frier BM . Morbidity of hypoglycemia in type 1 diabetes. Diabetes Res Clin Pract. 2004;65:S47‐S52. 15315871 10.1016/j.diabres.2004.07.008

[13] Sicherer SH , Simons FER . Quandaries in prescribing an emergency action plan and self‐injectable epinephrine for first‐aid management of anaphylaxis in the community. J Allergy Clin Immunol. 2005;115(3):575‐583. 15753907 10.1016/j.jaci.2004.12.1122

[14] Sicherer SH , Simons F , Mahr TA , et al. Epinephrine for first‐aid management of anaphylaxis. Pediatrics. 2017;139(3):e20164006. 28193791 10.1542/peds.2016-4006

[15] Simons FER . First‐aid treatment of anaphylaxis to food: focus on epinephrine. J Allergy Clin Immunol. 2004;113(5):837‐844. 15131564 10.1016/j.jaci.2004.01.769

[16] Simons KJ , Simons FER . Epinephrine and its use in anaphylaxis: current issues. Curr Opin Allergy Clin Immunol. 2010;10(4):354‐361. 20543673 10.1097/ACI.0b013e32833bc670

[17] Pearson T . Glucagon as a treatment of severe hypoglycemia. Diabetes Educ. 2008;34(1):128‐134. 18267999 10.1177/0145721707312400

[18] Valentine V , Newswanger B , Prestrelski S , Andre AD , Garibaldi M . Human factors usability and validation studies of a glucagon autoinjector in a simulated severe hypoglycemia rescue situation. Diabetes Technol Ther. 2019;21(9):522‐530. 31219349 10.1089/dia.2019.0148 PMC6708285

[19] Yale J‐F , Dulude H , Egeth M , et al. Faster use and fewer failures with needle‐free nasal glucagon versus injectable glucagon in severe hypoglycemia rescue: a simulation study. Diabetes Technol Ther. 2017;19(7):423‐432. 28556672 10.1089/dia.2016.0460 PMC5563859

[20] Wood JP , Traub SJ , Lipinski C . Safety of epinephrine for anaphylaxis in the emergency setting. World J Emerg Med. 2013;4(4):245. 25215127 10.5847/wjem.j.issn.1920-8642.2013.04.001 PMC4129903

[21] Brown AF , McKinnon D , Chu K . Emergency department anaphylaxis: a review of 142 patients in a single year. J Allergy Clin Immunol. 2001;108(5):861‐866. 11692116 10.1067/mai.2001.119028

[22] Amin A , Lau L , Crawford S , Edwards A , Pacaud D . Prospective assessment of hypoglycemia symptoms in children and adults with type 1 diabetes. Can J Diabetes. 2015;39:26‐31. 26541488 10.1016/j.jcjd.2015.09.086

[23] Simmons JH , McFann KK , Brown AC , et al. Reliability of the diabetes fear of injecting and self‐testing questionnaire in pediatric patients with type 1 diabetes. Diabetes Care. 2007;30(4):987‐988. 17392558 10.2337/dc06-1553

[24] Mohr DC , Cox D , Merluzzi N . Self‐injection anxiety training: a treatment for patients unable to self‐inject injectable medications. Mult Scler J. 2005;11(2):182‐185. 10.1191/1352458505ms1146oa 15794392

[25] Elman N , Ho Duc HL , Cima MJ . An implantable MEMS drug delivery device for rapid delivery in ambulatory emergency care. Biomed Microdevices. 2009;11(3):625‐631. 19169826 10.1007/s10544-008-9272-6

[26] Joo H , Lee Y , Kim J , et al. Soft implantable drug delivery device integrated wirelessly with wearable devices to treat fatal seizures. Sci Adv. 2021;7(1):eabd4639. 33523849 10.1126/sciadv.abd4639 PMC7775752

[27] Prescott JH , Lipka S , Baldwin S , et al. Chronic, programmed polypeptide delivery from an implanted, multireservoir microchip device. Nat Biotechnol. 2006;24(4):437‐438. 16531991 10.1038/nbt1199

[28] Farra R , Sheppard NF Jr , McCabe L , et al. First‐in‐human testing of a wirelessly controlled drug delivery microchip. Sci Transl Med. 2012;4(122):122ra21. 10.1126/scitranslmed.3003276 22344516

[29] Belverud S , Mogilner A , Schulder M . Intrathecal pumps. Neurotherapeutics. 2008;5(1):114‐122. 18164490 10.1016/j.nurt.2007.10.070 PMC5084133

[30] Bazaka K , Jacob MV . Implantable devices: issues and challenges. Electronics. 2012;2(1):1‐34.

[31] Cowan DB , McGowan FX Jr . A paradigm shift in cardiac pacing therapy? In Am Heart Assoc. 2006;114:986‐988. 10.1161/CIRCULATIONAHA.106.644799 PMC1570537 16952993

[32] Takeuchi H , Ishida M , Furuya A , Todo H , Urano H , Sugibayashi K . Influence of skin thickness on the in vitro permeabilities of drugs through Sprague‐Dawley rat or Yucatan micropig skin. Biol Pharm Bull. 2012;35(2):192‐202. 22293349 10.1248/bpb.35.192

[33] Gibney MA , Arce CH , Byron KJ , Hirsch LJ . Skin and subcutaneous adipose layer thickness in adults with diabetes at sites used for insulin injections: implications for needle length recommendations. Curr Med Res Opin. 2010;26(6):1519‐1530. 20429833 10.1185/03007995.2010.481203

[34] Lee SH , Kim CR , Cho YC , et al. Magnetically actuating implantable pump for the on‐demand and needle‐free administration of human growth hormone. Int J Pharm. 2022;618:121664. 35292393 10.1016/j.ijpharm.2022.121664

[35] Perry L , Karp F , Hauch K , Ratner BD . Explanted pacemakers: observations of the long‐term foreign body response. Interface. 2007;4:11.

[36] Kim CR , Cho YC , Lee SH , et al. Implantable device actuated by manual button clicks for noninvasive self‐drug administration. Bioeng Translat Med. 2022;e10320. https://doi/10.1002/btm2.10320 10.1002/btm2.10320 PMC9842066 36684080

[37] Lee SH , Lee YB , Kim BH , et al. Implantable batteryless device for on‐demand and pulsatile insulin administration. Nat Commun. 2017;8(1):1‐10. 28406149 10.1038/ncomms15032 PMC5399301

[38] Fromer L . Prevention of anaphylaxis: the role of the epinephrine auto‐injector. Am J Med. 2016;129(12):1244‐1250. 27555092 10.1016/j.amjmed.2016.07.018

[39] Christiansen MP , Cummins M , Prestrelski S , Close NC , Nguyen A , Junaidi K . Comparison of a ready‐to‐use liquid glucagon injection administered by autoinjector to glucagon emergency kit for the symptomatic relief of severe hypoglycemia: two randomized crossover non‐inferiority studies. BMJ Open Diabetes Rese Care. 2021;9(1):e002137. 10.1136/bmjdrc-2021-002137 PMC8499286 34620618

[40] Sicherer SH , Simons FER , Allergy SO . Immunology, self‐injectable epinephrine for first‐aid management of anaphylaxis. Pediatrics. 2007;119(3):638‐646. 17332221 10.1542/peds.2006-3689

[41] Young MC , Muñoz‐Furlong A , Sicherer SH . Management of food allergies in schools: a perspective for allergists. J Allergy Clin Immunol. 2009;124(2):175‐182.e4. 19493563 10.1016/j.jaci.2009.04.004

[42] Simons FER . Anaphylaxis, killer allergy: long‐term management in the community. J Allergy Clin Immunol. 2006;117(2):367‐377. 16461138 10.1016/j.jaci.2005.12.002

[43] Newswanger B , Prestrelski S , Andre AD . Human factors studies of a prefilled syringe with stable liquid glucagon in a simulated severe hypoglycemia rescue situation. Expert Opin Drug Deliv. 2019;16(9):1015‐1025. 31475853 10.1080/17425247.2019.1653278

[44] Simons FER , Lieberman PL , Read EJ Jr , Edwards ES . Hazards of unintentional injection of epinephrine from autoinjectors: a systematic review. Ann Allergy Asthma Immunol. 2009;102(4):282‐287. 19441598 10.1016/S1081-1206(10)60332-8

[45] Carlson ML , Neff BA , Link MJ , et al. Magnetic resonance imaging with cochlear implant magnet in place: safety and imaging quality. Otol Neurotol. 2015;36(6):965‐971. 25931165 10.1097/MAO.0000000000000666

[46] Sidambe AT . Biocompatibility of advanced manufactured titanium implants—a review. Materials. 2014;7(12):8168‐8188. 28788296 10.3390/ma7128168 PMC5456424

[47] Elias C , Lima J , Valiev R , Meyers M . Biomedical applications of titanium and its alloys. J Miner. 2008;60(3):46‐49.

[48] Farid R , Binz K , Emerson JA , Murdock F . Accuracy and precision of the SynchroMed II pump. Neuromodulation. 2019;22(7):805‐810. 30889307 10.1111/ner.12934

[49] Rauck R , Deer T , Rosen S , et al. Accuracy and efficacy of intrathecal administration of morphine sulfate for treatment of intractable pain using the Prometra® programmable pump. Neuromodulation. 2010;13(2):102‐108. 21992782 10.1111/j.1525-1403.2009.00257.x

[50] Cosgun MS , Cosgun C . Predictors of shoulder limitations and disability in patients with cardiac implantable electronic devices: importance of device size. Pacing Clin Electrophysiol. 2021;44(12):1979‐1986. 34624142 10.1111/pace.14378

[51] Hamdan HMA . Measurements of ELF electromagnetic fields in Jordan exposure limits and recommendations. Dirasat, Eng Sci. 2013;39(1):109‐118.

[52] Kleiner LW , Wright JC , Wang Y . Evolution of implantable and insertable drug delivery systems. J Control Release. 2014;181:1‐10. 24548479 10.1016/j.jconrel.2014.02.006

[53] O'Keefe A , Clarke A , Pierre YS , et al. The risk of recurrent anaphylaxis. J Pediatr. 2017;180:217‐221. 27743592 10.1016/j.jpeds.2016.09.028

[54] Yakubovich N , Gerstein HC . Serious cardiovascular outcomes in diabetes: the role of hypoglycemia. Circulation. 2011;123(3):342‐348. 21263007 10.1161/CIRCULATIONAHA.110.948489

[55] Sheybani R , Gensler H , Meng E . A MEMS electrochemical bellows actuator for fluid metering applications. Biomed Microdevices. 2013;15(1):37‐48. 22833156 10.1007/s10544-012-9685-0 PMC3755886

[56] Tan T , Watts SW , Davis RP . Drug delivery: enabling technology for drug discovery and development. iPRECIO® micro infusion pump: programmable, refillable, and implantable. Front Pharmacol. 2011;2:44. 21863140 10.3389/fphar.2011.00044 PMC3149148

[57] Pope JE , Deer TR . Intrathecal drug delivery for pain: a clinical guide and future directions. Pain Manage. 2015;5(3):175‐183. 10.2217/pmt.15.12 25971641

[58] Donnelly RF , Yen M . Epinephrine stability in plastic syringes and glass vials. Can J Hosp Pharm. 1996;49:2.

[59] Hoellein L , Holzgrabe U . Ficts and facts of epinephrine and norepinephrine stability in injectable solutions. Int J Pharm. 2012;434(1–2):468‐480. 22613065 10.1016/j.ijpharm.2012.05.017

[60] Frokjaer S , Otzen DE . Protein drug stability: a formulation challenge. Nat Rev Drug Discov. 2005;4(4):298‐306. 15803194 10.1038/nrd1695

[61] Mendes GC , Brandao TR , Silva CL . Ethylene oxide sterilization of medical devices: a review. Am J Infect Control. 2007;35(9):574‐581. 17980234 10.1016/j.ajic.2006.10.014

[62] GhavamiNejad A , Li J , Lu B , et al. Glucose‐responsive composite microneedle patch for hypoglycemia‐triggered delivery of native glucagon. Adv Mater. 2019;31(30):1901051. 10.1002/adma.201901051 31165524

[63] Ji HB , Kim S‐N , Lee SH , et al. Soft implantable device with drug‐diffusion channels for the controlled release of diclofenac. J Control Release. 2020;318:176‐184. 31838204 10.1016/j.jconrel.2019.12.022

