# Supplementary material for: Implantable device with magnetically rotating disk for needle‐free administrations of emergency drug
Source: Bioeng Transl Med. 2023 Feb 1;8(3):e10479. doi: 10.1002/btm2.10479 (PMC10189441; doi:10.1002/btm2.10479)
Supplement: Supplementary file 1 — Data S1: Supporting Information [file BTM2-8-e10479-s001.pdf]

Supporting Information

Implantable Device with Magnetically-rotating Disk for Needle-free Administrations of Emergency Drug

Cho Rim Kim, Jae Hoon Han, Min Ji Kim, Myoung Ju Kim, Se-Na Kim, Yong Chan Cho, Han Bi Ji, Chang Hee Min, Cheol Lee, Young Bin Choy

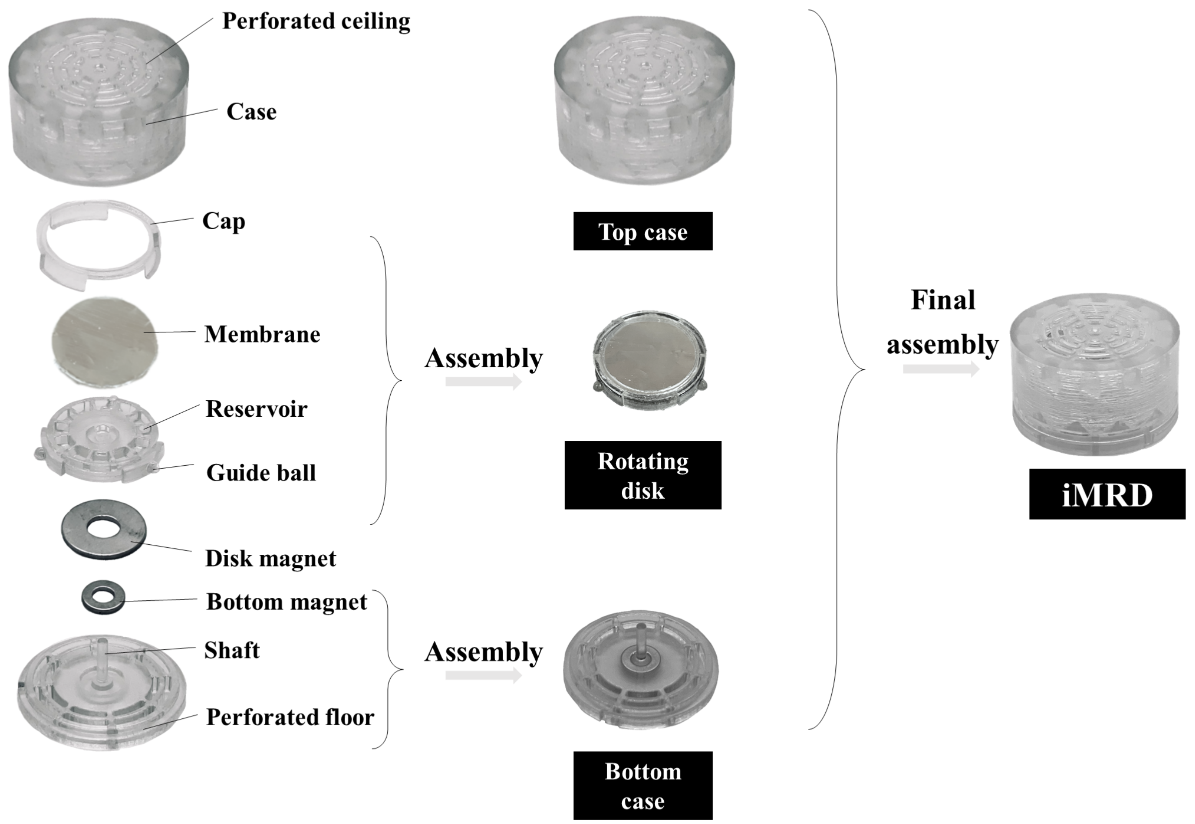

Figure S1. Optical images of the constituent parts and assembly procedure for the device.

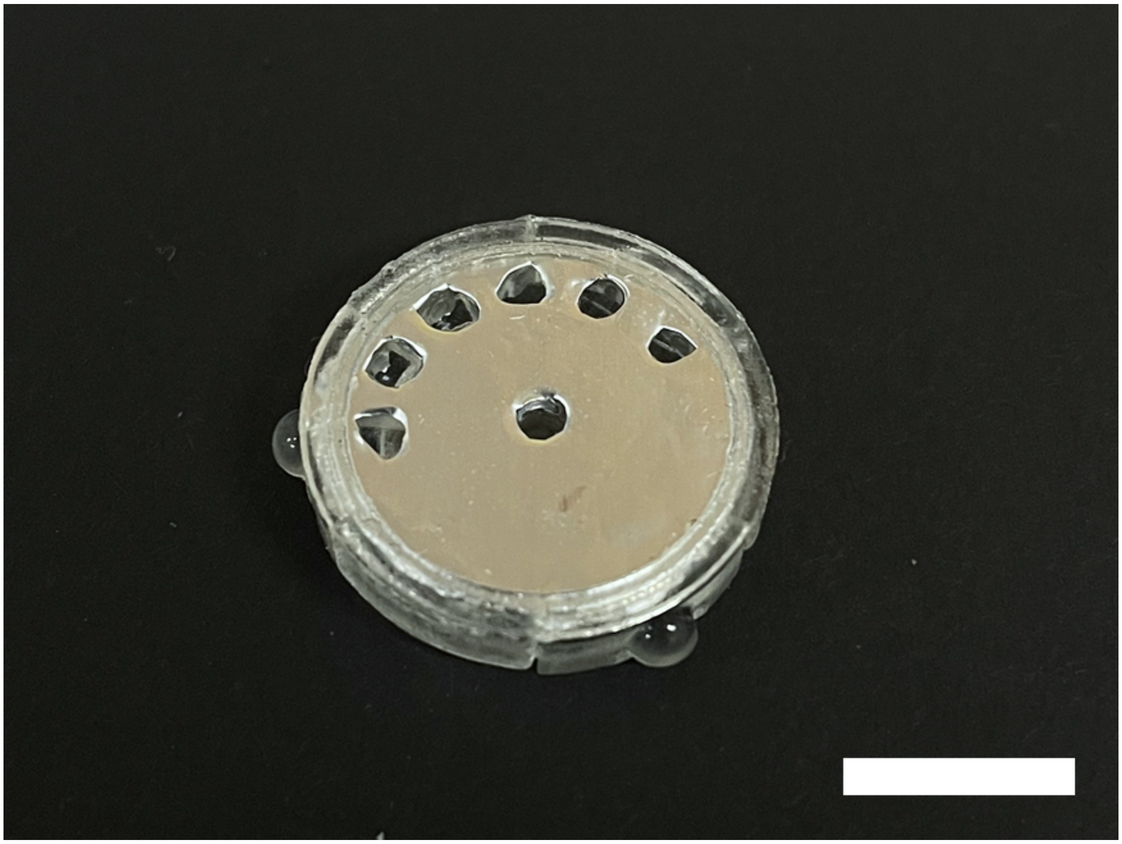

Figure S2. Optical image of the rotating disk disassembled from the device after six consecutive actuations. Only the designated reservoirs were open, and the rest six were intact. Scale bar, 1 cm.

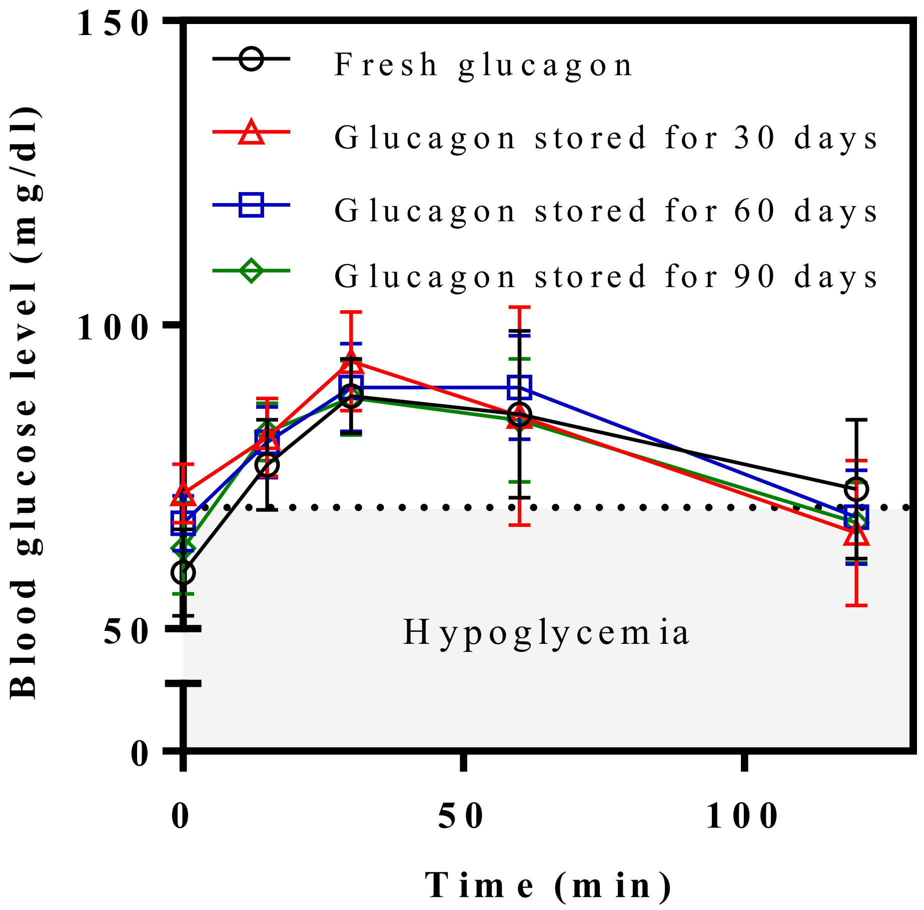

Figure S3. Biological activity of glucagon in the reservoirs of the iMRD incubated at 37 °C for 30, 60, and 90 days. To test this, the animals (Wistar rats) were fasted overnight, and the ones with a blood glucose level of < 70 mg/dl were selected as hypoglycemic model. After each incubation time period, glucagon in a reservoir (20 μg) was fully dissolved in 100 μl distilled water. For comparison, a fresh glucagon solution of the same dose was also prepared. The resulting glucagon solution was subcutaneously injected to the animal, after which, a blood glucose level was measured using a glucometer. For each type of the glucagon solutions, four animals were tested. Data is presented in the form of mean ± standard deviation (SD).

Supplementary Table 1. Pharmacokinetic parameters of epinephrine.

Implantation period	Day 15		Day 30		Day 45		Day 60	
Animal group	Epi-inj	Epi-iMRD	Epi-inj	Epi-iMRD	Epi-inj	Epi-iMRD	Epi-inj	Epi-iMRD
T_{max} (min)	30	30	30	30	30	30	30	30
C_{max} (pg/ml)	1185.09 ± 145.11	1129.69 ± 152.87	1086.14 ± 221.85	1023.94 ± 226.12	1054.88 ± 146.31	913.11 ± 92.11	1026.02 ± 140.99	939.52 ± 122.67
AUC (ng/ml min)	110.53 ± 3.64	111.30 ± 10.32	107.27 ± 6.28	106.58 ± 3.56	107.19 ± 5.72	105.38 ± 15.00	99.48 ± 10.53	102.14 ± 6.16

Supplementary Table 2. Pharmacokinetics parameters of glucagon.

Implantation period	Day 15		Day 30		Day 45		Day 60	
Animal group	Glu-inj	Glu-iMRD	Glu-inj	Glu-iMRD	Glu-inj	Glu-iMRD	Glu-inj	Glu-iMRD
T_{max} (min)	15	15	15	15	15	15	15	15
C_{max} (pg/ml)	106.11 ± 17.05	92.90 ± 7.57	97.89 ± 14.96	94.48 ± 8.98	99.86 ± 15.29	97.72 ± 9.26	104.51 ± 11.86	92.16 ± 14.75
AUC (pg/ml min)	7871.07 ± 703.40	7513.14 ± 349.22	7815.26 ± 329.36	7693.13 ± 935.39	7431.89 ± 519.74	7318.29 ± 558.38	6920.88 ± 698.71	7020.40 ± 225.52

Supplementary Table 3. Actuations of the iMRD according to the distance between the external magnet and iMRD. The iMRD could be actuated at a distance of up to 3 mm, which was greater than the thickness of human skins (~ 2 mm)[1].

Distance between the external magnet and iMRD (mm)	Actuation ability *
0	Y
1	Y
2	Y
3	Y
4	N

*Y: Reservoir opened; N: Reservoir not opened

Movie S1. Working principle of the iMRD.

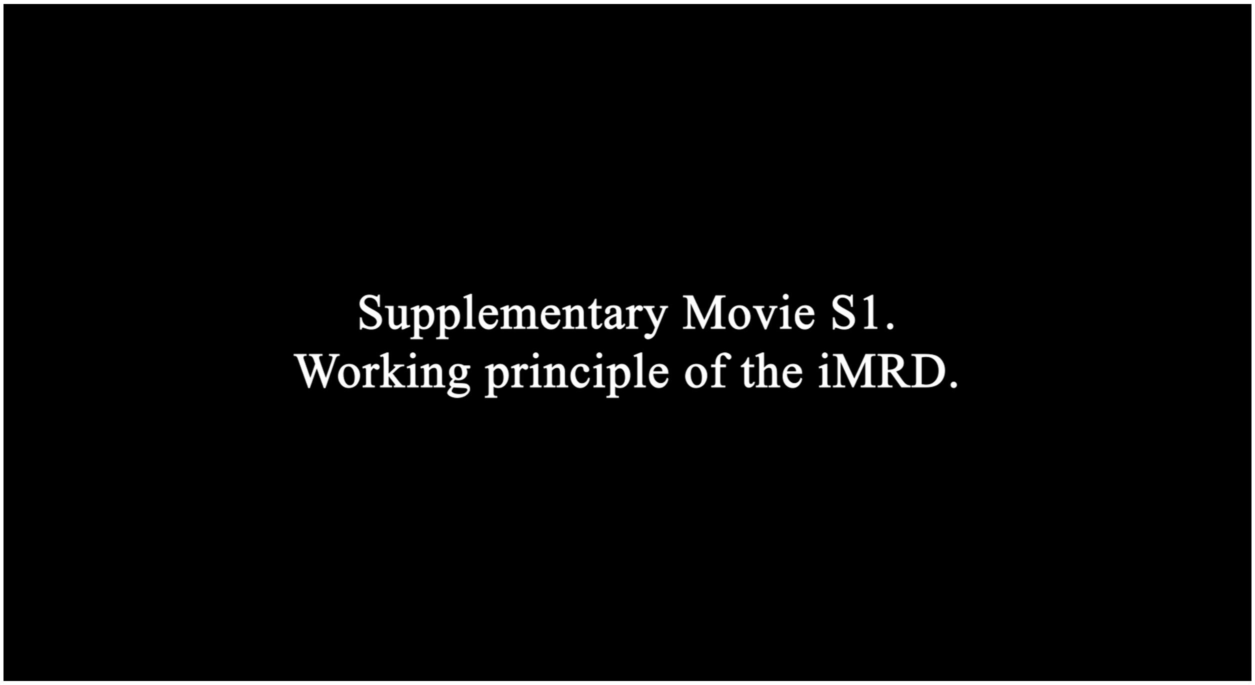

Movie S2. Membrane after iMRD actuations.

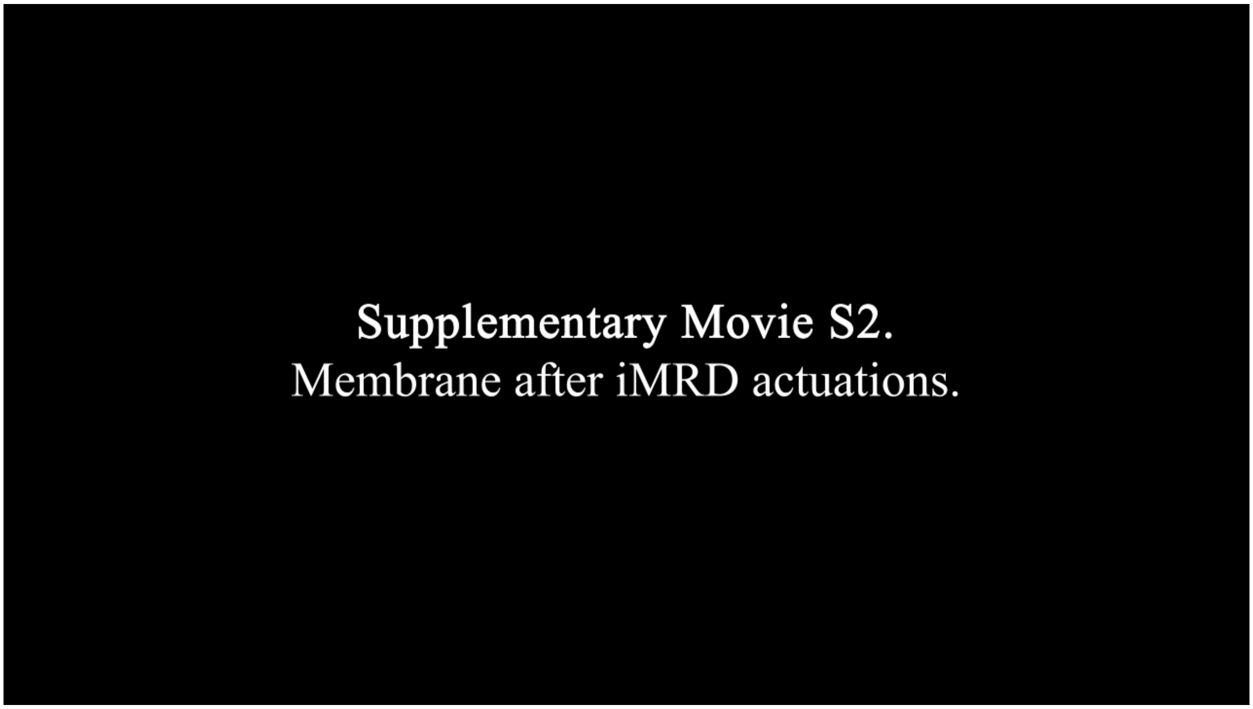

Reference

1. Gibney, M. A.; Arce, C. H.; Byron, K. J.; Hirsch, L. J., Skin and subcutaneous adipose layer thickness in adults with diabetes at sites used for insulin injections: implications for needle length recommendations. *Current medical research and opinion* **2010,** 26, (6), 1519-1530.
